# Normative Processing Needs Multiple Levels of Explanation: From Algorithm to Implementation

**DOI:** 10.1177/17456916231187393

**Published:** 2023-07-28

**Authors:** Todd Vogel, Patricia L. Lockwood

**Affiliations:** 1 Centre for Human Brain Health, School of Psychology, University of Birmingham; 2 Institute for Mental Health, School of Psychology, University of Birmingham; 3 Centre for Developmental Science, School of Psychology, University of Birmingham

**Keywords:** comparative psychology, learning, motivation/goals/reward, neuroscience, norm psychology, prosocial, reward, social cognition

## Abstract

Norms are the rules about what is allowed or forbidden by social groups. A key debate for norm psychology is whether these rules arise from mechanisms that are domain-specific and genetically inherited or domain-general and deployed for many other nonnorm processes. Here we argue for the importance of assessing and testing domain-specific and domain-general processes at multiple levels of explanation, from algorithmic (psychological) to implementational (neural). We also critically discuss findings from cognitive neuroscience supporting that social and nonsocial learning processes, essential for accounts of cultural evolution, can be dissociated at these two levels. This multilevel framework can generate new hypotheses and empirical tests of cultural evolution accounts of norm processing against purely domain-specific nativist alternatives.

Humans are guided by and enforce norms or rules upon one another. Whereas some are enshrined in law (e.g., do not steal), others operate more informally, such as how one greets other people. A key debate is whether the cognitive or mental processes underlying norms are domain-specific, genetically inherited modules (Fehr & Schurtenberger, 2018; Henrich & Muthukrishna, 2021; Kelly & Davis, 2018) or domain-general psychological and neural processes generated through social learning (Heyes, 2022). A new proposal draws from both nativist and domain-general accounts and places “cultural evolution” as central to norm psychology. In this proposal, norms are shaped by a distinctively human, domain-specific cognitive process that is learned through domain-general associative-learning mechanisms (Heyes, 2022). This novel cultural evolution of norm psychology (CENP) framework incorporates aspects of both domain-general and domain-specific processes while remaining nonnativist.

Here we argue that to empirically examine the CENP account against others, one must understand the processes involved at multiple levels of explanation—those that are algorithmic (psychological) and those that are implementational (neural). That is, in line with influential frameworks of information-processing systems (Marr, 1982), multiple levels of explanation must be assessed and tested to delineate the domain-specific and domain-general processes involved in norm processing (Lockwood, Apps, & Chang, 2020). For example, to understand bird flight, researchers cannot simply study feathers. They must know that the goal of the bird is to fly (computation), which it achieves by flapping (algorithm), and which is implemented in its feathers (implementation). Likewise, if researchers are to understand norm processing, they need to know not only that the goal is to engage in normative behavior but also the algorithm by which people achieve it (the psychological or mental processes) and the way it is implemented (its neural basis; Lockwood, Apps, & Chang, 2020).

We have previously discussed how this Marrian framework (Lockwood, Apps, & Chang, 2020) might be useful in resolving debates surrounding whether social learning is specifically social or arises from domain-general associative learning mechanisms (Catmur et al., 2009; Cook et al., 2014; Heyes, 2012). Drawing from research in cognitive neuroscience, there is evidence that the same algorithms (associative learning) can guide both social and nonsocial learning but that the implementation is realized in partially distinct brain areas (Apps et al., 2016; Behrens et al., 2008; Corlett et al., 2022; Lindström et al., 2018; Lockwood et al., 2016; Lockwood & Wittmann, 2018; Olsson et al., 2020; Sul et al., 2015).

Applying this framework to understand “disorders of normative processing” provides a clear empirical test of the need for multiple levels. Psychopathy—a developmental disorder preceded by “limited prosocial emotions” in childhood or adolescence—is characterized by norm violation, lack of empathy and guilt, and antisocial behaviour and impulsivity (Blair, 2013, 2017; Pauli & Lockwood, 2023). Several studies have shown that diagnosed psychopathy and psychopathic traits in the general population are linked with a reduction in associative-learning abilities (Blair, 2013, 2017; Pauli & Lockwood, 2023), consistent with a domain-general algorithmic impairment. However, evidence suggests that when social- and nonsocial-learning abilities are manipulated independently, social learning (learning how to benefit others or following social advice) is related to psychopathic traits, whereas tracking of nonsocial reward history is not (Brazil et al., 2013; Cutler et al., 2021).

Therefore, the same algorithm, associative learning, underlies people’s ability to learn what helps others and themselves but might be differentially implicated in core aspects of psychopathy. Indeed, at the implementational level, areas of the brain involved in processing social information have been shown to be affected in psychopathy with areas associated to nonsocial processing intact (Blair, 2013; Lockwood, 2016; Pauli & Lockwood, 2023). Thus, it is critical for experimental designs to keep one level constant in order to assess whether there is domain generality or specificity at the other level (Lockwood, Apps, & Chang, 2020). Distinguishing between multiple levels of explanation allows empirical testing of how and whether domain-general or domain-specific processes necessary for normative behavior operate, and this should be considered in any norm-processing account.

A second important aspect in an empirical framework of norm psychology is an appreciation of the basic building blocks for norms. Heyes (2022) proposed that a “cognitive gadget” that is culturally learned and domain specific is central for norm psychology. The cognitive-gadget version contrasts with theories that are innate and domain-general or innate and domain-specific. Again, we suggest that multiple levels of explanation must be considered when distinguishing these possibilities empirically.

Cultural evolutionary accounts assert that humans are “ultrasocial” (Henrich & Muthukrishna, 2021), that there is something distinctively human about normative behavior. However, we argue that the basic building blocks for norms are clear across many species. It is well accepted that organisms can process rewards and punishment or simply positive and negative action-outcome associations, which are essential for any form of learning or decision-making (Schultz, 2016). When one interrogates basic norm behavior, the same processes are involved but instead occur in a social context. Prosocial behaviors are simply actions chosen that help or avoid harming another agent (Lockwood et al., 2016) and are evident in some form across many species (Chen & Hong, 2018). Studies in rodents have shown that they avoid actions that harm others (Hernandez-Lallement et al., 2020). This effect is selectively abolished by inactivation of the anterior cingulate cortex (ACC; Hernandez-Lallement et al., 2020), which also disrupts observational fear learning while leaving classical conditioning intact (Jeon et al., 2010). Inactivation of the amygdala, by contrast, affects both observational learning and classical fear learning (Jeon et al., 2010). These are clear examples of possible specificity at algorithm and/or implementation for the basic foundations of norms.

One recent study provides a particularly interesting test of dissociation at the implementational level of norm processing. Basile and colleagues (2020) required macaque monkeys to learn that three different visual cues were associated with juice rewards to the monkey themselves (“self”), another monkey (“other”), or neither monkey (“neither”). They found all monkeys exhibited prosocial preferences in that they had a greater tendency to learn to reward other over neither. After lesioning the ACC, all monkeys retained the prosocial preferences they had demonstrated with the preoperatively learned cues. However, none of the ACC-lesioned monkeys were able to acquire prosocial preferences for a new set of cues, although their general learning abilities for self were still intact (Basile et al., 2020; Lockwood, O’Nell, & Apps, 2020). Therefore, damage at the implementational level to the ACC selectively affected the learning of new norms even though the algorithm, associative learning, was the same.

In summary, accounts of normative processing need to be able to explain the mechanisms by which normative processing operates, whether they are domain-general or domain-specific or involve elements of both within a cultural-learning framework. We argue that key to resolving this challenge will be an appreciation that cognitive and neural processes can be either domain-general and/or domain-specific at multiple levels of explanation. Revealing the algorithmic and implementational mechanisms can be done through careful experimental design and parallel theoretical frameworks that account for multiple levels.

## Transparency

*Action Editor:* Daniel Kelly

*Editor:* Interim Editorial Panel
